# The Unstable Cervical Disc

**Published:** 1961-04

**Authors:** D. M. Jones

**Affiliations:** Consultant Orthopaedic Surgeon, Winford and Southmead Hospitals


### THE UNSTABLE CERVICAL DISC

BY

D. M. JONES, M.CH.(ORTH.), F.R.C.S.

*Consultant Orthopaedic Surgeon, Winford and Southmead Hospitals*

The modern practice of orthopaedics is increasingly concerned with the treatment of painful disabilities of spinal origin. The frequency of these complaints was recently revealed in a record maintained at Southmead Hospital; 50 per cent of all new patients referred to the Orthopaedic Department during a period of fifteen months sought relief for symptoms whose source could be traced centrally to the spine. Amongst the patients with spinal disorders, the lesion was located in the lumbar spine in 51 per cent, and in the cervical spine in 41 per cent—the latter group accounting for one fifth of all the subjects.

With the exception of rheumatoid arthritis, inflammatory and neoplastic diseases are rare in the cervical spine. The usual cause of disability is cervical spondylosis, a term used to describe a degenerative condition primarily affecting the discs and articular facets. The posterior facets undergo a true osteoarthritis with the formation of osteophytes occasionally encroaching on the intervertebral foramina. The condition is common beyond the fifth decade when it is often associated with disc changes at various levels. Elderly patients, however, only occasionally complain of severe and persistent pain, their comparative freedom from discomfort being due at least in part to their having passed beyond the more strenuous period of their lives.

The more disabling syndromes are encountered among younger age groups, the term "younger" here embracing all ages from adolescence to middle life. The chief cause is the premature degeneration of a cervical disc, a transformation usually limited before the age of fifty to one or two of the structures. The condition is not necessarily painful, indeed it is often discovered accidentally. From the clinical standpoint many discs undergo considerable change while still retaining much of their original strength and stability. Though always a potential source of weakness and disability, they may continue "silent" provided an adjacent nerve root is not irritated by the marginal proliferation of scar tissue and osteophytes. In contrast to these more stable forms, and progressing to the ultimate extreme through all gradations in severity, are the discs that undergo a comparatively rapid and profound change in their internal structure. Losing their normally firm and elastic outline they are left weak and unstable. Abnormal stresses develop along their sensitive margins; movement and the loss of protective muscle tone during fatigue now give rise to pain locally or referred across the neck and shoulder (Kellgren 1960). The unstable condition is further indicated by spasm and fatigue of muscles, headaches and minor degrees of giddiness consequent on the loss of smooth and balanced movements at the cervical segments. Giddiness was a marked feature of one case described here; its relation to spondylosis at the upper cervical levels has been noted by others (Ryan and Cope, 1955).

Discomfort in the throat may also be related to instability at a cervical disc. The syndrome usually affects women and is sometimes attributed to disorders of a functional or nervous nature. Choking sensations are common, though the patients are aware of no real obstruction to swallowing. Frequent complaints are of feeling throttled and suffocated; the throat often feels constricted. The sensation of a lump may be simulated. These sensations probably arise from stimuli originating along the antero-lateral margins of the disc concerned, augmented possibly by spasm of muscles situated towards the front and sides of the throat. Stabilizing the weak disc has relieved patients on a number of occasions.

**House-wife, aged 50.** Aching across neck and shoulders, posterior headaches, stiffness and tiredness of neck. Paraesthesia of both hands in morning. X-rays revealed loss in depth at the fifth-sixth disc with reversal of the normal cervical curve. During the year previous to these complaints the patient had been unusually busy changing her home. She became aware at this time of the sensation of a lump in the throat; the symptom developed sufficiently to require examination by a throat surgeon, but no abnormality was found. The sensation was absent on lying down but became apparent soon after rising in the morning, and was always worse towards the end of a day.

**Spinster, aged 35,** a music teacher. Eight months history of brachial neuralgia into left arm approximating to distribution of seventh root. Neck stiffness and fatigue with posterior headaches for a longer period. Degeneration of the sixth–seventh disc seen in the X-ray. For at least a year previous to the onset of arm pain this patient had been aware of discomfort in the throat, mostly of a choking, suffocating nature. Sensation of a lump sometimes present just above the sternum. Symptoms always worse at end of day. Her chest had been X-rayed because of this trouble. Full plaster immobilization for three months gave only temporary relief.

Grafting of sixth–seventh disc July 1957. Some arm pain has persisted following operation but not as severe as previously. She has, however, lost all discomfort in the neck and throat. With her neck strengthened she no longer feels "on the verge of collapse at the end of a day's work".

The treatment of the cervical disc syndrome has hitherto remained almost exclusively conservative, the methods employed ranging from mobilization of the neck on the one hand to complete rest on the other, the two forms of treatment sometimes alternating in the same case. Stretching may relieve neuralgia in the more stable neck, but it is of little value where the trouble is due mainly to instability. Rest is required in the latter instance and is achieved in the majority of cases by plaster immobilization of neck, plastic collars, or comfortable soft supports for the less severe cases. The results obtained are not altogether satisfactory. The adult vertebral disc lacks the natural gift of healing. Rest abates the symptoms, but once all restraint is removed the constant movements of the neck not infrequently lead to a return of the symptoms, in the more intractable cases pursuing a chronically relapsing course extending over years.

Recent publications indicate a developing interest in the surgical treatment of cervical spondylosis. The majority of the papers are from neurosurgical sources and in consequence are largely concerned with the release of the cord and nerve roots from compression by advanced spondylotic change. Except in the most urgent cases it appears that such procedures are not wholly justified by the results (Campbell and Phillips 1960). They have, in any case, little bearing on the common orthopaedic problems where strength and stability in the neck with freedom from root irritation are the usual requisites. While it appears likely that orthopaedic surgeons have grafted persistently painful cervical discs on occasions, only a few references can be found to the attainment of these aims by operative means. Smith and Robinson (1958) described fourteen patients treated by grafting weak discs and the freeing of sensitive roots. Frykholm (1947) modified the operation of root decompression, removing the minimum of bone to avoid weakening the spine. Knight (1955) practised similar methods and has recently (1959) introduced the use of acrylic splints inserted deep to the spinal muscles.

Over twenty cases have been operated on in Bristol during the last four years. The measures employed have in the main been the grafting of unstable discs with decompression of painful roots. Change in a single disc offers the most convenient opportunities for surgery. A successful graft permanently relieves all local pain and may also abolish irritation of an adjacent root.

**Male, aged 43.** Three years history of pain across neck and shoulders, with neck stiffness and headaches. Symptoms often severe and influenced by posture. Some pain into proximal part of arms. X-rays revealed degeneration of the sixth–seventh disc. This patient had been treated by full plaster immobilization on two occasions, with only temporary relief. Grafting of the sixth–seventh disc, June 1958. Since the operation patient has remained free of all discomfort and regained a full range of neck movements. He has returned to playing cricket after a lapse of nearly three years.

**House-wife, aged 37.** Old degeneration of sixth–seventh disc activated by a fall. Eighteen months history of left brachial neuralgia with pain across neck and shoulders, stiffness and headaches. Choking and strangling sensations when tired. Unable to ride in bus as vibration brought on pain. Large osteophytes encroaching on the intervertebral foramina. Plaster immobilization for three months gave only temporary relief.

Grafting sixth–seventh disc, August, 1957.

Since operation patient has remained free of all discomfort, able to participate to the full in work and pleasure.

Where pain referred into the arm has been severe and persistent the bone overlying the sensitive root has usually been removed. Frykholm (1957) incised the meninges surrounding the exposed root, freeing the nerve from kinks and adhesions. Knight (1955) claimed satisfactory results following the removal of bone alone, a modification adhered to in this series. The procedure appears to contribute towards the relief of pain.

**Male, aged 44, a clerk.** Three years history of brachial neuralgia approximating to seventh root distribution. Symptoms consistent with instability, with pain at back of neck spreading across shoulders, neck stiffness and headaches. Spent week-ends resting in order to recuperate for the ensuing week's work. X-rays revealed minimal changes at the sixth–seventh interval. Operation October 1958. Decompression right seventh root with grafting of disc. Patient has since remained free of all discomfort. Full neck movements. Is once more gardening and decorating his home.

**House-wife, aged 50.** Three years history of pain and stiffness in neck and across shoulders, becoming more severe, with headaches and mild giddiness. Right brachial neuralgia for eighteen months. Constriction of throat when tired. Marked loss of rotation of neck due to pain; would allow not more than one-third the normal range when turning to the left. X-rays revealed degeneration of fifth–sixth disc with reversal of normal cervical curve at this level. Immobilization gave only temporary relief. Operation October 1959. Decompression of right sixth root with graft. Since operation patient has remained free of all discomfort and regained a full range of neck movement.

**House-wife, aged 50.** A tired, haggard-looking woman referred from the psychiatric clinic. General picture one of anxiety and depression. Five years history of brachial neuralgia, worse on right side, with headaches, neck stiffness, giddiness and choking sensations. X-rays: degeneration of fifth–sixth and sixth–seventh discs. Background story of stress and unhappiness in home. Outlook not apparently favourable for any form of treatment. Operation November 1958. Decompression of right sixth and seventh roots with grafting fifth–sixth and sixth–seventh laminae. This patient has done surprisingly well. No discomfort in neck or throat; pain in arm on one occasion only. She appears rested and well nourished, and content with the result. Since operation her practitioner has been consulted on one occasion only for this complaint—a passing attack of neuralgia following over-use of arm.

Fibrosis of the root sleeves, peridural adhesions and caudal displacement of the roots have been described in the intervertebral foramina adjacent to a degenerated disc (Frykholm, 1951). They were noted in three cases in this series and considered responsible for some persistence of neuralgia after operation, though in only one case was the pain sufficient to be a nuisance. These conditions develop gradually and are indicative of disc change of some duration. Brachial neuralgia may be an early symptom of instability, occurring before the development of adhesions and osteophytes. In one patient recently operated on, arm pain was the first symptom, due, it was believed, to traction on the dura following abnormal movement at the disc.

Root decompression was performed in two elderly patients, with no attempt to stabilize the spine. Both patients had extensive osteoarthritis of the facets and complained of persistent arm pain. One patient obtained considerable relief but was left aware of discomfort in the neck. The second case was complicated by disc changes at various levels, and the patient experienced only temporary relief. Both patients might well have benefited from some form of durable support. The difficulty in these and many other patients is to define the segment requiring support and to avoid, for obvious reasons, grafting indefinite lengths of the cervical spine. Acrylic splints, should these fulfil the claims of their innovator, may help to solve some of these problems. Acrylic inlays have been used in three recent cases.

**House-wife, aged 50.** First treated in 1952 for fibrositis of neck and shoulders. Seen again in 1957; condition had steadily progressed; complains now of bilateral brachial neuralgia, headaches, giddiness and neck pain.

Hysterectomy 1957; repair of hiatus hernia 1958. Returned for further treatment in 1959. Complaints as before but worse. Giddiness was now a feature; because of it the patient had lost confidence and refused to venture outside her home. She held her neck stiffly; when looking round did so by turning her body, holding her neck more or less still. Often felt the need to support her head in her hands. X-rays revealed degeneration at the third–fourth, fourth-fifth and fifth–sixth discs, with osteophytes projecting into the foramina. Operation May 1960. Decompression right fifth and sixth roots with acrylic inlay over laminae of the third to sixth vertebrae. Though it is too early to assess the result the patient remains free of discomfort in neck and arms. She has also recovered a good range of neck movement.

The operative measures here described have imposed no great strain on the patient. Autogenous grafts of iliac bone take well, and over short lengths prove more durable in the neck than at other levels of the spine. The post-operative regime was early reduced to two weeks recumbency in a firm wool collar, followed by the wearing of a plaster collar for a further ten weeks, all wounds healing rapidly. One patient died suddenly of pulmonary embolus on the sixteenth day after the operation. Following this misfortune patients have been treated more energetically, the last four cases of grafting becoming ambulant from the fourth day onwards.

The surgical treatment of these "orthopaedic" examples of cervical spondylosis is likely to be regarded with increasing favour in the future. In suitable cases a complete cure is obtained, enabling patients to return to full activity in both work and pleasure. In others a worth-while measure of permanent relief follows, terminating an otherwise protracted and recurrently painful condition. Such results are not obtained by other methods in the treatment of the unstable cervical disc. A further point of some importance is that grafting may safeguard the future of the cord by preventing the gradual enlargement of cartilage and bone formations at the margins of the disc and by maintaining the alignment of the neck. For these reasons operations are sometimes worth considering but it is, for obvious reasons, first necessary to decide with complete assurance the correct level of the lesion. Herein so often lies the difficulty. The development of a comparatively simple and innocuous technique capable of demonstrating weak discs would do much to facilitate the management of these cases.

I am indebted to Mr. D. C. Berry, Lecturer in Prosthetic Surgery at the University of Bristol, for expert guidance in the preparation of acrylic splints.

### REFERENCES

Campbell, A. M. G. and Phillips, D. G. (1960). *Brit. Med. J.*, *ii*, 481
Frykholm, R. (1947). *Jour. of Neurosurgery*, **4**, 403.
Frykholm R. (1951). *Act. Chirurg. Scand.*, **101**, 457.
Kellgren, J. H. (1939). *Clin. Science*, **4**, 35
Knight, G. C. )1955). *Proc. Roy. Soc. Med.*, **48**, 595.
Knight, G. C. (1959). *Lancet*, *ii*, 147.
Ryan, G. M. S. and Cope, S. (1955), *Lancet*, *ii*, 355
Smith, G. W. and Robinson, R. A. (1958). *Journ. of Bone and Joint Surgery*, **40-A**, 681.

